# Radiotherapy vs surgery for T1‐2N0M0 laryngeal squamous cell carcinoma: A population‐based and propensity score matching study

**DOI:** 10.1002/cam4.1525

**Published:** 2018-05-07

**Authors:** Cheng Zhan, Xiaodong Yang, Xinmao Song, Li Yan

**Affiliations:** ^1^ Department of Thoracic Surgery Zhongshan Hospital Fudan University Shanghai China; ^2^ Department of Radiation Oncology Eye & ENT Hospital Fudan University Shanghai China

**Keywords:** laryngeal squamous cell carcinoma, radiotherapy, SEER, surgery

## Abstract

There are conflicting reports about whether radiotherapy or surgery is optimal for early‐stage laryngeal squamous cell carcinoma (LSCC), although both have recently been recommended. Patients with T1‐2N0M0 LSCC in the population‐based SEER database who underwent radiotherapy or surgery were reviewed. Propensity score matching was used to eliminate the baseline variations. After matching, 1913 pairs of patients were included. Overall, patients who received radiotherapy had worse cancer‐specific survival than patients with surgery. After stratification, the survival in patients who received radiotherapy was worse with respect to the following characteristics: ≤60 years of age; T1a glottis cancer; well‐differentiated tumors; and with married status. In other patients, survival outcomes were similar in patients who received radiotherapy and underwent surgery. Our results indicate that radiotherapy is not preferable in early‐stage LSCC patients who are ≤60 years of age, have T1a glottis cancer or well‐differentiated tumors, or are married. In other patients, both radiotherapy and surgery are comparable. However, our results cannot be a reference before controlled, prospective trials are performed.

## 1 INTRODUCTION

As one of the most common cancers worldwide, laryngeal carcinoma accounts for estimated 160 000 new cases and 90 000 deaths every year, of which >95% are squamous cell carcinomas.^1^, ^2^, ^3^ Depending on the disease stage at diagnosis, the primary management strategies for patients with laryngeal squamous cell carcinoma (LSCC) mainly consist of surgery and/or radiotherapy. Although the cure rate for early‐stage (T1‐2N0M0) LSCC is favorable, and ranges between 60% and 100% in several studies, the optimal treatment strategy has not been determined.^4^ According to the latest NCCN guidelines, surgery or definitive radiotherapy can be used for curing T1‐2N0M0 LSCC. Several studies have demonstrated improved survival with radiation over surgery.^5^ Survival benefit in patients with early‐stage LSCC undergoing surgery has also been reported in a number of other studies.^6^, ^7^, ^8^ Due to the conflicting reports, further exploration of the optimal treatment method for early‐stage LSCC is warranted.

The Surveillance, Epidemiology and End Results (SEER) program is a large population‐based source for cancer statistics, which gives detailed information on incidence, prevalence, and survival from specific geographic areas and compiled reports on all of these plus cancer mortality.^9^ Using cases in SEER, we attempted to determine whether the radiation or surgery could be an optimal treatment regimen for patients with T1‐2N0M0 LSCC in this study. Meantime, propensity score matching was used to reduce bias caused by clinical characteristic variance between groups, which may contribute to the existing conflicting consequences. However, it is noteworthy that our results cannot be a reference before controlled, prospective trials are performed.

## 2 METHODS

### 2.1 Data selection

**Figure 1:**
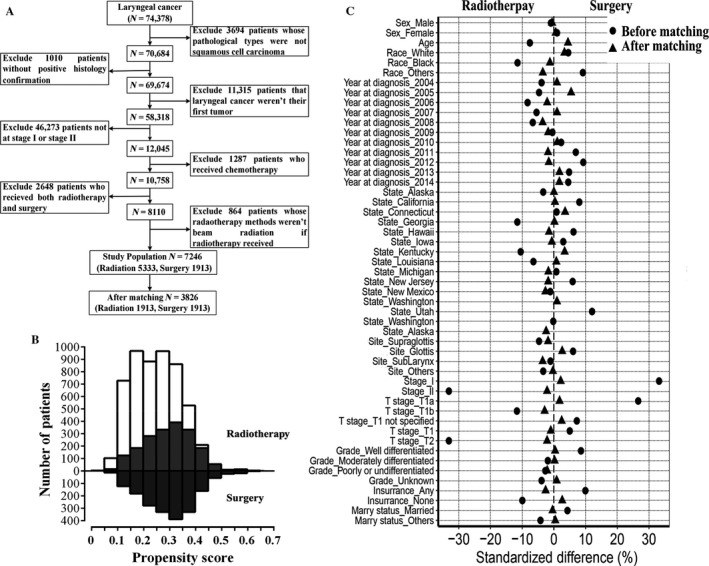
A, Flow diagram of selecting process. B, Mirror histograms of propensity scores for patients with radiotherapy and with surgery. Matched patients are presented in dark color. C, Standardized differences of baseline variables between patients with radiation and with surgery before and after propensity score matching

SEER (Incidence—SEER 18 Regs Custom Data with additional treatment fields, Nov 2016 Sub, 1973—2014 varying) data were obtained via the SEER*Stat software (version 8.3.4; http://seer.cancer.gov/seerstat/). To acquire sufficient data from the database, the selection process is shown in Figure 1. Briefly, patients with labeled primary sites C32.0‐Glottis, C32.1‐Supraglottis, C32.2‐Subglottis, C32.3‐Laryngeal cartilage, C32.8‐Overlapping lesion of larynx, or C32.9‐Larynx NOS were included. Exclusion criteria were as follows: (1) not SCC; (2) without positive histology confirmation; (3) not the first tumor; (4) not stage I or stage II; (4) chemotherapy received; (5) underwent both radiotherapy and surgery; (6) not beam radiation if radiotherapy administered. Conversion from the old version to the seventh AJCC TNM staging system was performed manually. All patients who met the inclusion and exclusion criteria were divided into radiotherapy and surgery groups according to the mode of therapy.

Clinical, pathologic, and sociodemographic characteristics, including age, gender, race, year of diagnosis, state, primary site, grade, T classification, TNM stage, insurance, and marital status at the time of diagnosis, were included in the analyses. Age was categorized by 10 years (≤50, 51‐60, 61‐70, 71‐80, >80 years of age at diagnosis). The LSCC cancer‐specific survival status and non‐LSCC cancer‐specific survival status were extracted from the variables “SEER cause‐specific death classification” and “SEER other cause of death classification” in SEER database. The LSCC cancer‐specific survival outcome attributed to SCC cancer‐specific deaths and survival time was censored at the date of lost follow‐up, the last contact data, or the date of death from other causes, whichever occurred first. The non‐LSCC cancer‐specific survival status was used for competing risk analyses.

### 2.2 Study design and statistical analysis

Statistical analyses were performed using IBM SPSS Statistics 24.0 (IBM, Inc., Armonk, NY) and R version 3.3.4 (R Foundation for Statistical Computing, Vienna, Austria).

The patients were divided into two groups according to the mode of treatment (radiotherapy vs surgery). The survival analysis was performed using a propensity score matching system to overcome patient selection bias among the baseline variables of the two groups.^10^, ^11^ Covariates thought to determine the choice of grouped patients were matched in the study as follows: age; gender; race; year of diagnosis; state; site; grade; T classification; TNM stage; insurance; and marital status at the time of diagnosis. The exposure for the propensity model was set as surgery.

Propensity scores were carried out using the “MatchIt” package.^10^, ^12^, ^13^ Matching results were obtained with the “nearest” matching method, and every case of the surgery group was matched to the control from the radiotherapy group with the closest propensity score. A standardized difference <10% is acceptable to assess the balance of covariates before and after matching.^14^

The baseline characteristics between the radiotherapy and surgery groups before and after matching were compared using Wilcoxon and chi‐square tests. Univariate and multivariate analyses were calculated using the Cox proportional hazards model. Only the variables with a *P*‐value <.05 in the univariate analyses entered into the multivariate analyses, while the multivariate analyses were performed with the backward stepwise (likelihood ratio) method and a threshold 0.10. The survival rates were estimated by the Kaplan‐Meier method, and log‐rank test was used for comparing survival curves after propensity score matching. Competing risk analyses were performed, as previously reported.^15^ A two‐tailed *P*‐value <.05 was considered statistically significant.

## 3 RESULTS

### 3.1 Patient characteristics

As shown in Figure 1A, LSCC accounted for approximately 95.0% (70 684/74 378) of laryngeal tumor cases in 1973‐2014 records of SEER database. Patients who were diagnosed between 1973 and 2003 were all excluded in the selection process as there was no TNM stage information of them. After rigorous selection, 7246 LSCC cases (5333 radiation vs 1913 surgery) were included in our research. The baseline demographics and clinical characteristics of all participants are summarized in Tables 1 and S1. Compared to the patients who underwent surgery, the patients who received radiotherapy were older (*P *=* *.005), had worse tumor differentiation (*P *=* *.010), had a higher T classification (*P *<* *.001) and TNM stage (*P *<* *.001), were more likely to be black (*P *<* *.001), and were less likely to have insurance (*P *<* *.001). Patients who underwent surgery represented an increasing proportion (*P *<* *.001) from 2004 to 2014. These variations in baseline characteristics may have a marked impact on survival outcomes.

**Table 1:** Patient characteristics according to the therapy status before and after propensity score matching

Characteristics	Before matching				After matching			
	Radiation	Surgery	SD (%)	*P* value	Radiation	Surgery	SD (%)	*P* value
Total number	5333	1913			1913	1913		
Sex								
Male	4482	1601	−0.958	.719	1605	1601	−0.567	.861
Female	851	312	0.958		308	312	0.567	
Age	65.4 ± 11.1	64.5 ± 12.1	−7.599	.005	64.7 ± 11.5	64.5 ± 12.1	0.132	.965
Race								
White	4453	1629	4.554	<.001	1606	1629	3.327	.496
Black	682	176	−11.49		183	176	−1.255	
Others	198	108	9.162		124	108	−3.505	
Year at diagnosis^a^				<.001				.838
State^a^				<.001				.960
Site								
Supraglottis	950	307	−4.709	.154	320	307	−1.836	.457
Glottis	4166	1541	6.021		1521	1541	2.616	
Sublarynx	38	12	−1.045		18	12	−3.557	
Others	179	53	−3.401		54	53	−0.317	
Grade								
Well differentiated	991	421	8.523	.010	418	421	0.379	.928
Moderately differentiated	2507	881	−1.916		879	881	0.210	
Poorly or undifferentiated	545	180	−2.723		192	180	−2.117	
Unknown	1290	431	−3.922		424	431	0.878	
Stage								
I	3601	1564	33.16	<.001	1548	1564	2.147	.507
II	1732	349	−33.16		365	349	−2.147	
T stage								
T1a	1795	891	26.59	<.001	874	891	1.783	.764
T1b	444	103	−11.66		116	103	−2.926	
T1 not specified	834	351	7.219		333	351	2.456	
T1	528	219	5.013		225	219	−0.979	
T2	1732	349	−33.16		365	349	−2.147	
Insurance status at diagnosis								
Any	3680	1406	9.939	<.001	1428	1406	−2.624	.417
None or unknown	1653	507	−9.939		485	507	2.624	
Marital status at diagnosis								
Married	3100	1152	4.255	.111	1156	1152	−0.427	.895
Others	2233	761	−4.255		757	761	0.427	

a Detailed data of year at diagnosis and state are listed in Table S1.

After matching based on propensity scores, 1913 pairs of patients were selected; one‐half were treated with radiotherapy and another half underwent surgery. There were no significant differences between the two groups, and all the *P* values for age, year of diagnosis, state, grade, stage, T classification, and insurance status had been greatly improved (Tables 1 and S1). The absolute values of standardized differences in matched variables were all <10%, suggesting that the variables were well balanced after matching. The matched groups had similar propensity score distributions, and the mirror histograms of propensity scores for patients are shown in Figure 1B,C.

### 3.2 Survival analyses

**Figure 2:**
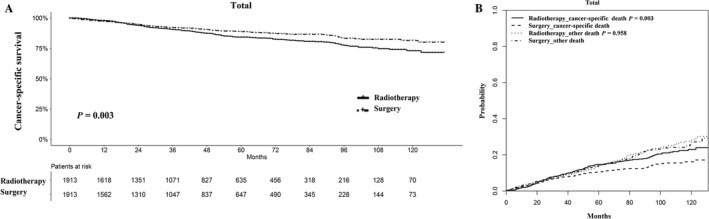
Survival analyses for patients with radiotherapy and with surgery. A, Kaplan‐Meier method; B, competing risk analysis

The survival outcomes for the two groups of LSCC patients with T1‐2N0M0 tumors are shown in Figure 2. Patients who received radiation had a distinctly worse survival when compared with patients who underwent surgery; the five‐year cancer‐specific survival rates were 83.9 ± 1.1%% and 88.5 ± 0.9%, respectively (*P *=* *.003; Figure 2A). Competing risk analysis also indicated that the patients who received radiation had a higher risk of LSCC‐associated mortality (*P *=* *.003), while there was no significant difference in the probabilities of other causes of death (*P *=* *.958; Figure 2B).

We further examined the correlation between survival and other parameters. Univariate analyses revealed that age (*P *<* *.001), state (*P *<* *.004), site (*P *<* *.001), grade (*P *<* *.001), stage (*P *<* *.001), T classification (*P *<* *.001), and marital status (*P *<* *.001) were statistically significant predictors of LSCC‐specific survival in addition to therapy, as shown in Tables 2 and S2. No significant difference was demonstrated for gender (*P *=* *.572), race (*P *=* *.188), year of diagnosis (*P *=* *.286), and insurance status (*P *=* *.252). Based on multivariate analysis, therapy (*P *=* *.003), age (*P *<* *.001), grade (*P *=* *.016), T classification (*P *<* *.001), and marital status (*P *<* *.001) remained independent prognostic predictors for LSCC patients. The variables, site (*P *=* *.190) and stage (*P *=* *.636), were not significant predictors of survival based on multivariate analysis because they were not independent from the T classification.

**Table 2:** Results of univariate and multivariate analyses of cancer‐specific survival after matching

Characteristics	Univariate analysis			Multivariate analysis		
	HR	95% CI	*P* value	HR	95% CI	*P* value
Therapy			.003			.003
Radiation	Reference	Reference		Reference	Reference	
Surgery	0.746	0.615‐0.906	.003	0.741	0.610‐0.901	.003
Sex			.572	Not included		
Male	Reference					
Female	1.076	0.834‐1.389	.572			
Age	1.033	1.024‐1.042	<.001	1.039	1.030‐1.049	<.001
Race			.188	Not included		
White	Reference	Reference				
Black	1.232	0.916‐1.657	.168			
Others	0.774	0.488‐1.229	.278			
Year at diagnosis^a^			.286	Not included		
State^a^			.004			.113
Site			<.001			.190
Supraglottis	Reference			Reference		
Glottis	0.405	0.328‐0.501	<.001	0.711	0.490‐1.032	.072
Sublarynx	0.480	0.153‐1.508	.209	0.471	0.149‐1.491	.200
Others	1.113	0.720‐1.723	.630	1.033	0.661‐1.615	.886
Grade			<.001			.016
Well differentiated	Reference			Reference		
Moderately differentiated	1.436	1.089‐1.894	.010	1.239	0.936‐1.640	.134
Poorly or undifferentiated	2.331	1.663‐3.266	<.001	1.703	1.204‐2.408	.003
Unknown	1.196	0.867‐1.651	.275	1.113	0.804‐1.540	.519
Stage			<.001			.636
I	Reference			Reference		
II	1.921	1.556‐2.371	<.001	1.059	0.835‐1.343	.636
T stage			<.001			<.001
T1a	Reference			Reference		
T1b	1.513	0.970‐2.361	.068	1.502	0.961‐2.347	.074
T1 not specified	1.289	0.947‐1.755	.106	1.280	0.940‐1.743	.117
T1	3.040	2.315‐3.991	<.001	3.200	2.420‐4.230	<.001
T2	2.644	2.060‐3.394	<.001	2.618	2.031‐3.374	<.001
Insurance status at diagnosis			.052	Not included		
Any	Reference					
None or unknown	1.227	0.998‐1.509	.052			
Marital status at diagnosis			<.001			<.001
Married	Reference			Reference		
Others	1.521	1.256‐1.843	<.001	1.472	1.213‐1.787	<.001

a Detailed data of year at diagnosis and state are listed in Table S2.

**Figure 3:**
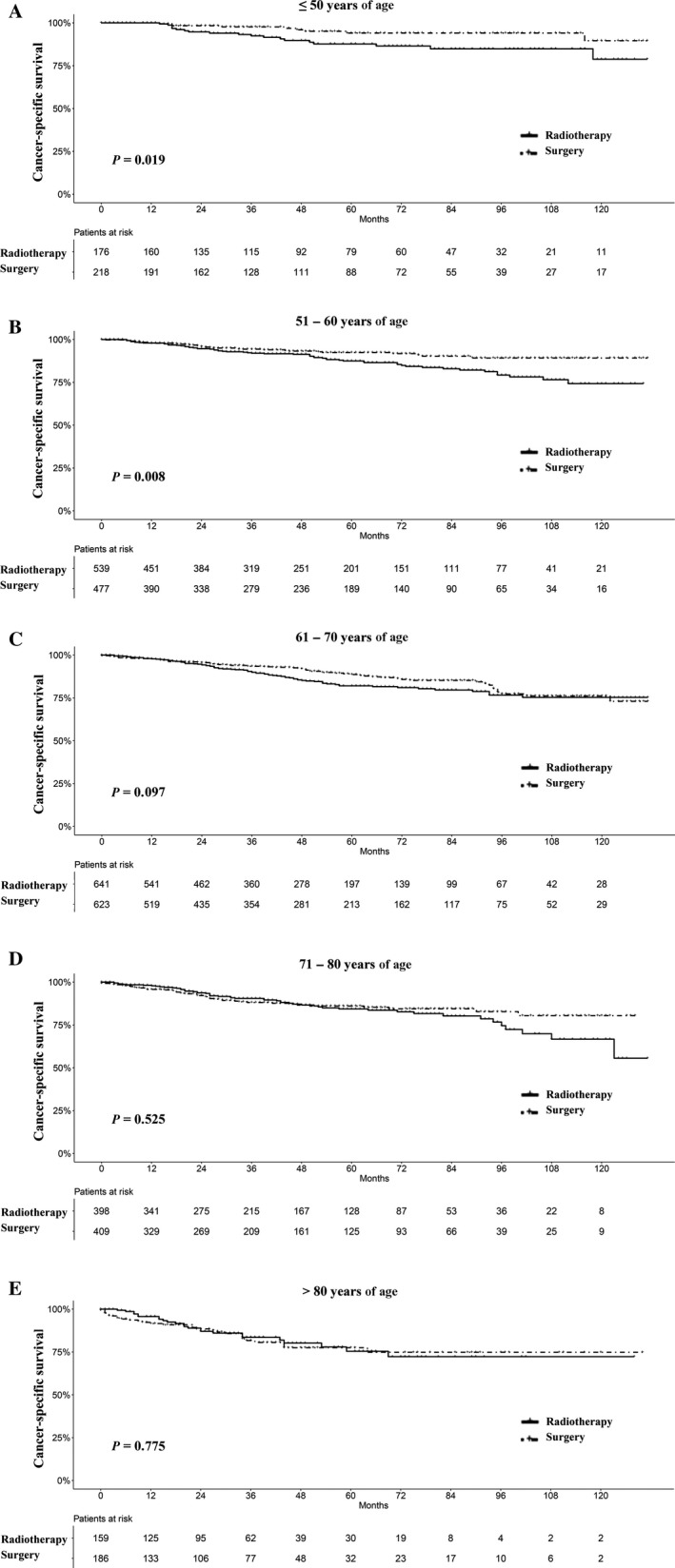
Survival analyses for patients with radiotherapy and with surgery stratified by age after matching. A, ≤50 years of age; B, 51‐60 years of age; C, 61‐70 years of age; D, 71‐80 years of age; and E, >80 years of age

To better characterize the impact of therapeutic approaches on survival of LSCC patients, we stratified the matched patients by variables which were significant based on multivariable analysis. As Figures 3 and S1 show, in patients ≤60 years of age, survival in the radiotherapy group was significantly worse than that of the surgery group. In patients >60 years of age, however, there was no significant difference in survival between the radiotherapy and surgery groups. The survival curves were almost overlapping, especially in patients >70 years of age.

**Figure 4:**
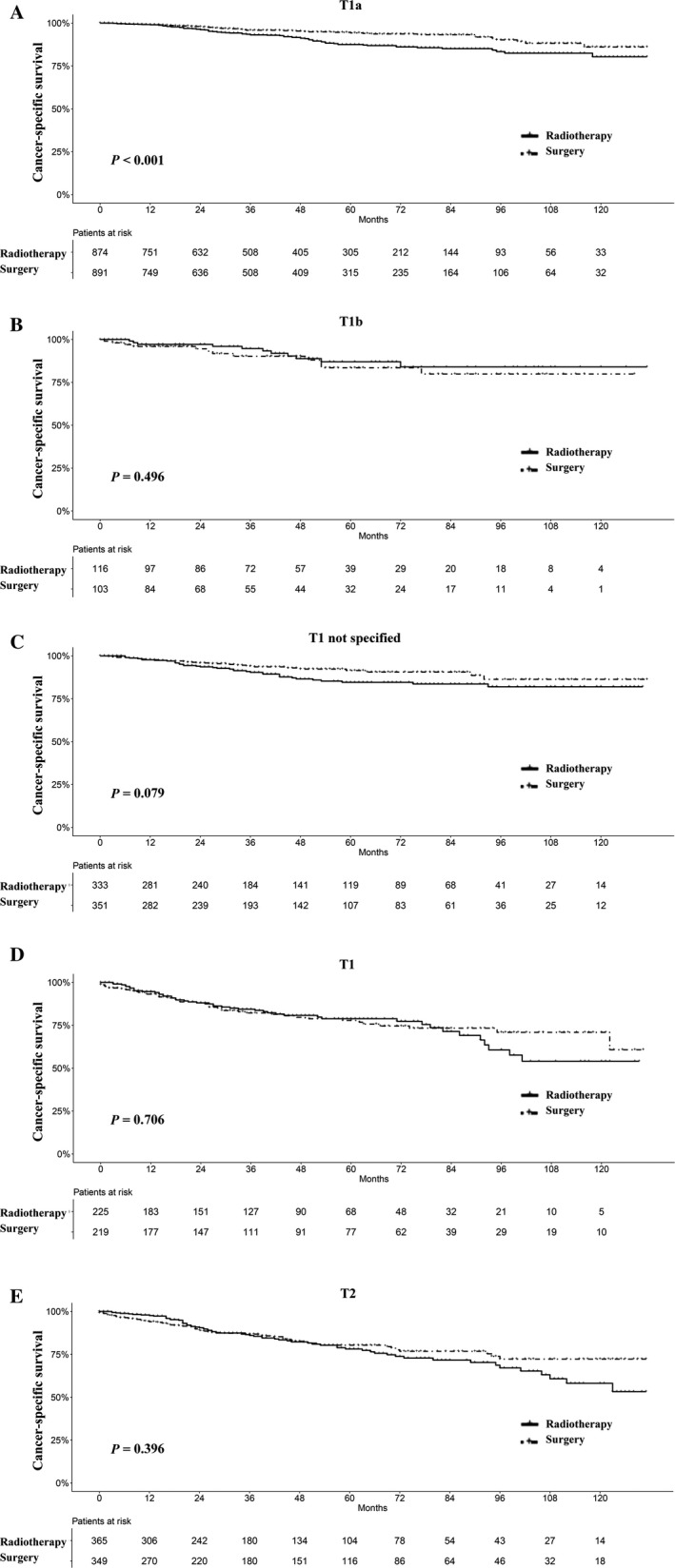
Survival analyses for patients with radiotherapy and with surgery stratified by T stage after matching. A, T1a; B, T1b; C, T1NOS; D, T1; and E, T2. T1a, T1b, and T1NOS are subsets of glottis cancer

**Figure 5:**
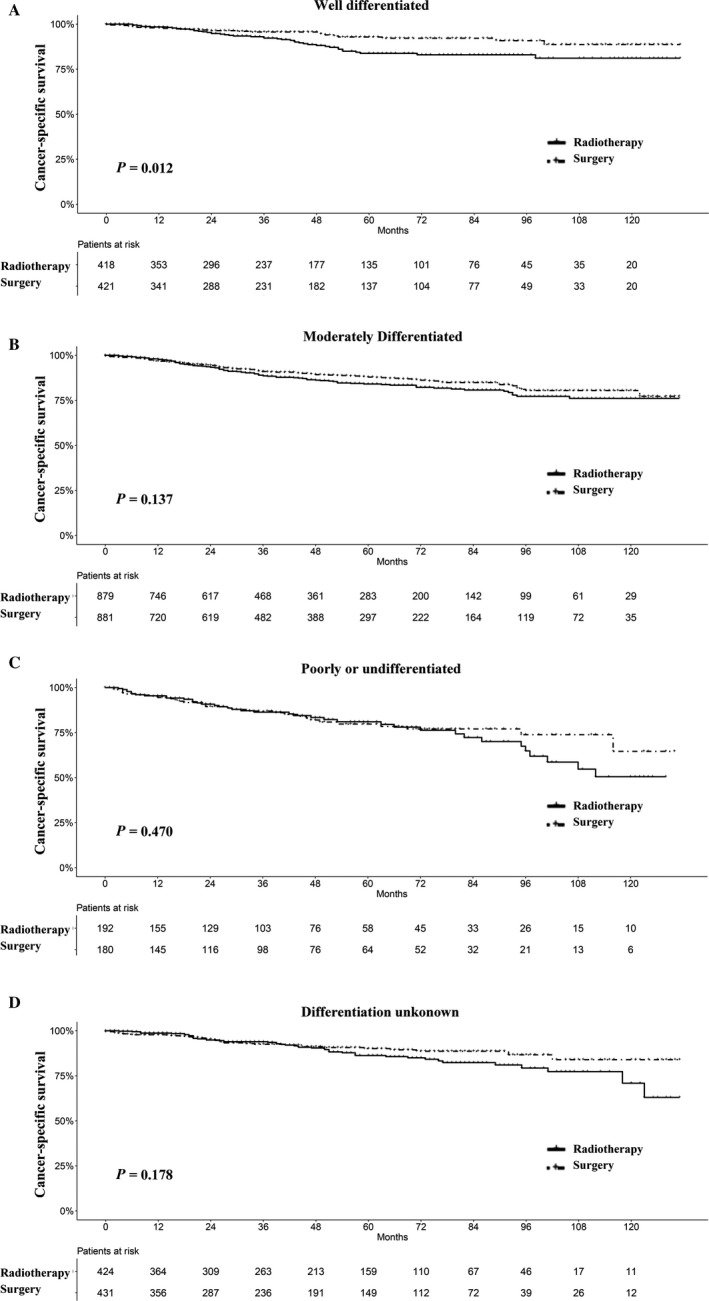
Survival analyses for patients with radiotherapy and with surgery stratified by differentiation after matching. A, Well differentiated; B, moderately differentiated; C, poorly or undifferentiated; and D, differentiation unknown

In the recent TNM stage system of laryngeal cancer, T1 classification of glottis cancer is divided into T1a, T1b, and T1 not specified. In our analyses, we found that only patients with stage T1a glottic cancer who underwent surgery had superior survival to radiotherapy, while there was no significant difference in T1b and T1 not specified glottic cancer, as shown in Figures 4 and S2. Neither T1 nor T2 supraglottis and subglottis squamous cell carcinomas had significant differences in survival. With respect to differentiation stage, radiotherapy had a comparable survival as surgery in moderately differentiated, poorly or undifferentiated tumors, but not in well‐differentiated LSCC patients (Figures 5 and S3).

Interestingly, our analyses showed that surgery had a preferable survival in patients who were married when diagnosed (Figure S4); however, in divorced, single, separated, widowed, or unmarried patients, there were no differences in survival between the radiotherapy and surgery groups.

As shown in Figures [Link], [Link], [Link], [Link], the results of competing risk analyses further validated the findings mentioned above after the fully consideration of other death causes.

In all, radiotherapy resulted in a significantly worse survival in LSCC patients with the following characteristics: ≤60 years of age, T1a glottis cancer, well‐differentiated tumors, or married (patients with either one characteristic accounted for 85.4% (6188/7246) of all T1‐2N0M0 LSCC patients in our cohort). Radiotherapy was not inferior to surgery for the treatment of all other LSCC patients.

## 4 DISCUSSION

In this study, we compared the survival of 7246 patients with early‐stage LSCC who underwent radiotherapy and surgery. Overall, our study indicated a hypothesis that patients with T1a stage of glottis cancer who were ≤60 years of age, married, or with well‐differentiated tumors treated with radiotherapy had worse survival outcomes than patients treated with surgery.

The present study had several limitations that should be noted. First, because the SEER database did not provide detailed information, we could not calculate the influence of factors, such as radiation technology, radiation dose, and surgery regimens. Different surgical or radiotherapy techniques have been adopted in the different institutions, which have great impact on patients’ survival. Second, in this analysis, we only focused on treatment mortality but not life quality as the data of life quality were not included in SEER database. Third, the patients included were from the USA; thus, the results might not be applicable to other populations. The last but not least, although the SEER database is population based and offers excellent follow‐up records, our study was retrospective and prospective studies with a larger randomized study cohort are needed to further validate our results.

Our study was based on the data obtained from SEER, a population‐based database. Population‐based studies may be misinterpreted as they provide comparative survival curves similar to curves found in reports of phase III trials. In fact, population‐based data should not be taken as reference studies for clinical decisions, because many biases may obscure their conclusions. In the case of the present study, it is clear from the data before matching that the two populations of patients treated with radiation or with surgery are not prognostically similar, with clear disadvantages for the radiation population.

Megwalu et al^16^ reported that patients with early‐stage laryngeal cancer treated with surgery have better survival outcomes than patients treated with radiotherapy. Our analyses revealed that patients with radiotherapy were older, had worse differentiated tumors, had higher‐stage tumors, were more likely to be black, and were less likely to have insurance. These variations in baseline characteristics may have a marked impact on survival outcomes. In the current study, we used propensity score matching to eliminate the potential bias caused by the variations in baseline characteristics, while all matched values were well balanced and radiotherapy obtained comparable survival outcomes in a number of patients. Propensity score matching has been frequently applied, and it is expected to play an increasingly important role in future clinical analyses.^17^, ^18^, ^19^ It is a useful statistical tool to generate hypotheses, but by no means may be taken as a substitute for proper randomization. In addition, a competing risk model was used in the current study to avoid the occurrence of cancer‐specific deaths hindered by other deaths.^15^

The optimal treatment for elderly people with LSCC is not well defined.^20^ In the current study, for laryngeal patients >60 years of age, radiotherapy produced comparable survival outcomes compared with surgery. Our results indicated that radiation therapy was closely effective in elderly LSCC patients.

Among LSCCs, glottic cancer is the most common subtype, accounting for 80% of LSCC patients in the current study. Several studies have concluded that surgery is associated with a higher survival in patients with LSCCs, while there are a number of studies that have reported the opposite results; however, most of these studies were based on <100 patients.^21^, ^22^, ^23^, ^24^ In a large meta‐analysis that included 562 participants treated with laser surgery and 706 participants treated with radiotherapy, the pooled analysis showed that laser surgery significantly improved the overall survival of patients with T1 glottic carcinoma group.^25^ In the current study, surgery yielded better survival than radiotherapy in patients with T1a stage glottis SCC, but not T1b, T1 not specified, or T2 stages. Our analysis showed that there is no statistically significant association between the mode of treatment and survival of early‐stage supra‐ and subglottic SCC, which is consistent with a previous report.^26^ The number of patients with T1‐2N0M0 subglottic cancer in our study was limited, perhaps because the vast majority of patients with subglottic cancer present with a locally advanced stage.^27^

Previous reports have revealed that patients with poorly differentiated LSCC fared less well than patients with better differentiated tumors.^28^, ^29^ In the current study, we also found that the grade of differentiation significantly influenced survival based on univariate and multivariate analyses. Compared to more differentiated cancer cells, less differentiated cells reproduce more and have a diminished ability to repair sublethal damage caused by radiotherapy, which will be inherited through cell division, thus accumulating damage to cancer cells. As a result, cells either die or reproduce more slowly.^30^ Perhaps this attribute is why LSCC surgery is superior to radiotherapy in patients with well‐differentiated tumors, but the survival is comparable in patients with moderately differentiated, poorly differentiated, or undifferentiated LSCC who undergo surgery or receive radiotherapy.

In the current study, we showed that marital status had a significant impact on the prognosis of LSCC patients as previously reported.^31^ Inverso et al^32^ also reported that marriage had a protective effect against metastatic presentation of laryngeal cancers. Our results showed that surgery had a better survival rate for married patients, while the survival of unmarried patients was similar whether treated with surgery and radiotherapy.

In our study, we only focused on treatment mortality but not voice outcomes or larynx preservations as these data were not recorded in SEER database. In a recent meta‐analysis, Huang et al^33^ reported that patients with radiotherapy may have the advantage of increased maximum phonation time and decreased fundamental frequency compared with patients undergoing laser surgery in T1a glottis cancer. However, Du et al^34^ reported that the acoustic voice analysis parameters of Fo values were significantly lower in radiotherapy group than those in laser surgery group in patients with early glottic cancer. Huang et al^35^ also reported that laser surgery may benefit from increased larynx preservation compared with radiotherapy. Now, there are still lacks of studies focused on the voice outcomes or larynx preservation of supraglottis or sublarynx cancer. More trials are still needed.

## 5 CONCLUSION

Our results indicate a hypothesis that radiotherapy is not a preferable option in early‐stage LSCC patients who are ≤60 years of age, have T1a glottis cancer or well‐differentiated tumors, or are married. In all other patients with early‐stage LSCC, radiotherapy will yield comparable survival outcomes. However, as the information of radiation and surgery from SEER database is not detailed enough, the study results cannot be a reference before controlled, prospective trials are performed.

## CONFLICT OF INTEREST

None declared.

## ETHICS STATEMENT

Ethics approval was exempted by the Ethics Committee of the Eye & ENT Hospital of Fudan University (Shanghai, China), as the SEER is a publicly available database, and data extracted from SEER were identified as nonhuman study.
