# Supplementary figures and images for: Radiotherapy vs surgery for T1‐2N0M0 laryngeal squamous cell carcinoma: A population‐based and propensity score matching study
Source: Cancer Med. 2018 May 7;7(7):2837–47. doi: 10.1002/cam4.1525 (PMC6051150; doi:10.1002/cam4.1525)

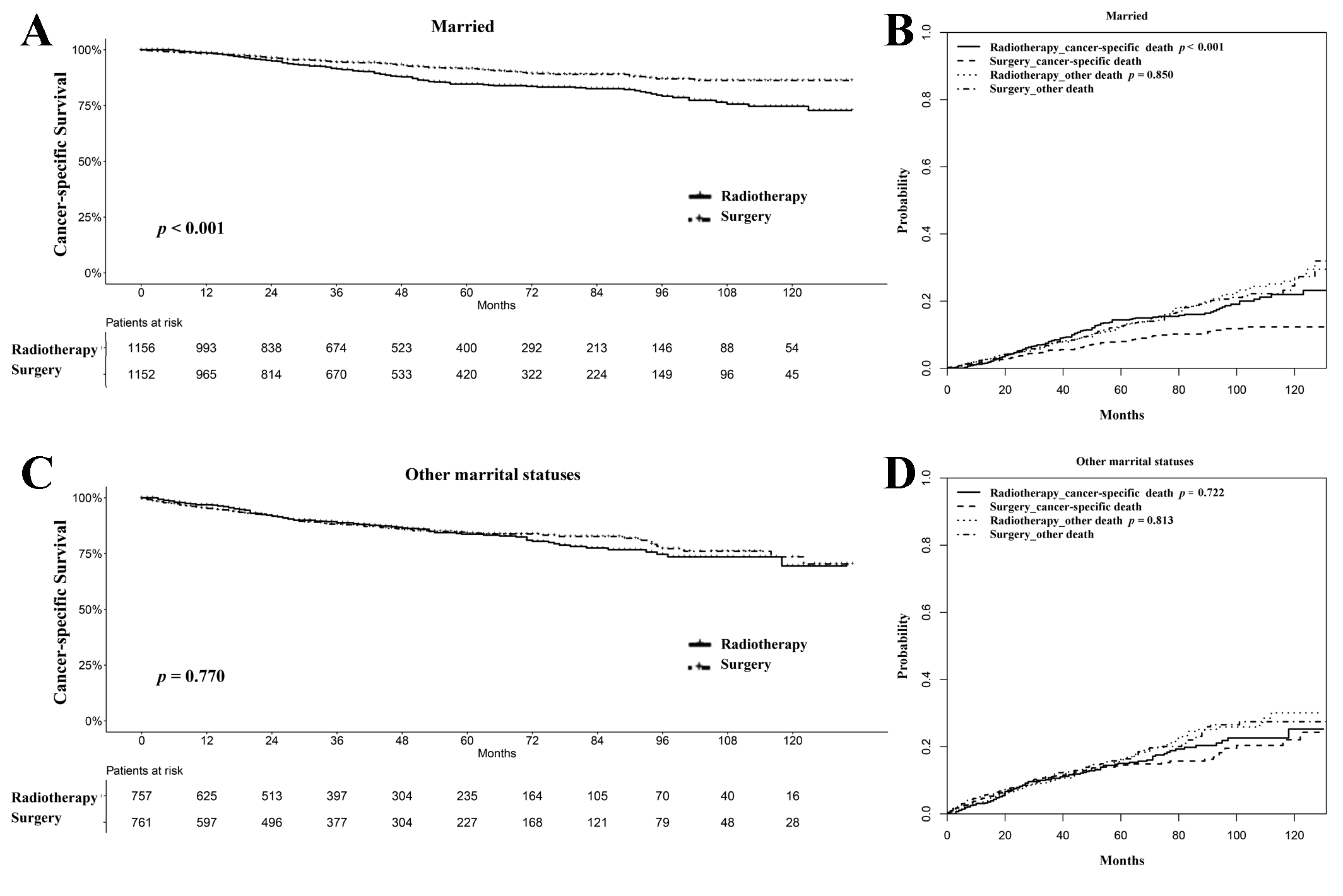

Supplement: Supplementary file 4 [file CAM4-7-2837-s004.tif]
