# Supplementary material for: Radiotherapy vs surgery for T1‐2N0M0 laryngeal squamous cell carcinoma: A population‐based and propensity score matching study
Source: Cancer Med. 2018 May 7;7(7):2837–47. doi: 10.1002/cam4.1525 (PMC6051150; doi:10.1002/cam4.1525)
Supplement: Supplementary file 5 [file CAM4-7-2837-s005.docx]

**Table S1** Characteristics of year at diagnosis and state of patients according to the therapy status before and after propensity score matching.

| **Characteristics** | **Before Matching** | | | |  | | **After Matching** | | | |
| --- | --- | --- | --- | --- | --- | --- | --- | --- | --- | --- |
|  | **Radiation** | **Surgery** | **SD(%)** | ***p* value** | | **Radiation** | | **Surgery** | **SD(%)** | ***p* value** |
| **Total number** | 5333 | 1913 |  |  | | 1913 | | 1913 |  |  |
| **Year at diagnosis** |  |  |  | <0.001 | |  | |  |  | 0.838 |
| 2004 | 485 | 153 | -3.923 |  | | 148 | | 153 | 0.971 |  |
| 2005 | 431 | 131 | -4.696 |  | | 106 | | 131 | 5.423 |  |
| 2006 | 536 | 147 | -8.331 |  | | 158 | | 147 | -2.123 |  |
| 2007 | 488 | 146 | -5.479 |  | | 141 | | 146 | 0.992 |  |
| 2008 | 531 | 154 | -6.665 |  | | 173 | | 154 | -3.553 |  |
| 2009 | 484 | 171 | -0.478 |  | | 181 | | 171 | -1.809 |  |
| 2010 | 499 | 192 | 2.297 |  | | 186 | | 192 | 1.051 |  |
| 2011 | 450 | 200 | 6.900 |  | | 211 | | 200 | -1.857 |  |
| 2012 | 469 | 222 | 9.297 |  | | 232 | | 222 | -1.616 |  |
| 2013 | 466 | 194 | 4.800 |  | | 184 | | 194 | 1.752 |  |
| 2014 | 494 | 203 | 4.509 |  | | 193 | | 203 | 1.716 |  |
| **State** |  |  |  | <0.001 | |  | |  |  | 0.960 |
| Alaska | 3 | 0 | -3.355 |  | | 0 | | 0 |  |  |
| California | 1572 | 635 | 8.020 |  | | 632 | | 635 | 0.333 |  |
| Connecticut | 288 | 107 | 0.847 |  | | 92 | | 107 | 3.532 |  |
| Georgia | 775 | 205 | -11.51 |  | | 204 | | 205 | 0.169 |  |
| Hawaii | 56 | 34 | 6.163 |  | | 38 | | 34 | -1.539 |  |
| Iowa | 295 | 119 | 2.930 |  | | 122 | | 119 | -0.646 |  |
| Kentucky | 581 | 150 | -10.49 |  | | 133 | | 150 | 3.396 |  |
| Louisiana | 543 | 159 | -6.460 |  | | 155 | | 159 | 0.762 |  |
| Michigan | 308 | 114 | 0.782 |  | | 122 | | 114 | -1.738 |  |
| New Jersey | 509 | 217 | 5.885 |  | | 228 | | 217 | -1.794 |  |
| New Mexico | 100 | 33 | -1.129 |  | | 40 | | 33 | -2.675 |  |
| Utah | 61 | 54 | 12.06 |  | | 51 | | 54 | 0.960 |  |
| Washington | 242 | 86 | -0.203 |  | | 96 | | 86 | -2.456 |  |

**Supporting Information**

**Table S1** Characteristics of year at diagnosis and state of patients according to the therapy status before and after propensity score matching.

**Table S2** Results of year at diagnosis and state in univariate and multivariate analyses of cancer-specific survival after matching.

**Figure S1** Competing risk analyses for patients with radiotherapy and with surgery stratified by age after matching (A) ≤ 50 years of age. (B) 51-60 years of age. (C) 61-70 years of age. (D) 71-80 years of age. (E) > 80 years of age.

**Figure S2** Competing risk analyses for patients with radiotherapy and with surgery stratified by T stage after matching (A) T1a. (B) T1b. (C) T1NOS. (D) T1. (E) T2. T1a, T1b, and T1NOS are subsets of glottis cancer.

**Figure S3** Competing risk analyses for patients with radiotherapy and with surgery stratified by differentiation after matching (A) Well differentiated. (B) Moderately differentiated. (C) Poorly or undifferentiated. (D) Differentiation unknown.

**Figure S4** Survival analyses for patients with radiotherapy and with surgery stratified by marital status. (A) Married patients, Kaplan-Meier method. (B) Married patients, competing risk analysis. (C) Patients at other marital status, Kaplan-Meier method. (D) Patients at other marital status, competing risk analysis.
