# Supplementary material for: Radiotherapy vs surgery for T1‐2N0M0 laryngeal squamous cell carcinoma: A population‐based and propensity score matching study
Source: Cancer Med. 2018 May 7;7(7):2837–47. doi: 10.1002/cam4.1525 (PMC6051150; doi:10.1002/cam4.1525)
Supplement: Supplementary file 6 [file CAM4-7-2837-s006.docx]

Table S2 Results of year at diagnosis and state in univariate and multivariate analyses of cancer-specific survival after matching.

| **Characteristics** | **Univariate analyses** | | |  | | **Multivariate analysis** | | |
| --- | --- | --- | --- | --- | --- | --- | --- | --- |
|  | **HR** | **95%CI** | ***p* value** | | **HR** | | **95%CI** | ***p* value** |
| **Year at diagnosis** |  |  | 0.286 | | Not included | |  |  |
| 2004 | Reference |  |  | |  | |  |  |
| 2005 | 1.155 | 0.794-1.679 | 0.451 | |  | |  |  |
| 2006 | 1.130 | 0.786-1.626 | 0.509 | |  | |  |  |
| 2007 | 0.910 | 0.609-1.360 | 0.647 | |  | |  |  |
| 2008 | 0.739 | 0.485-1.126 | 0.160 | |  | |  |  |
| 2009 | 0.885 | 0.591-1.324 | 0.552 | |  | |  |  |
| 2010 | 1.032 | 0.695-1.533 | 0.876 | |  | |  |  |
| 2011 | 0.843 | 0.545-1.304 | 0.444 | |  | |  |  |
| 2012 | 0.670 | 0.406-1.105 | 0.117 | |  | |  |  |
| 2013 | 0.949 | 0.516-1.744 | 0.866 | |  | |  |  |
| 2014 | 0.192 | 0.026-1.406 | 0.104 | |  | |  |  |
| **State** |  |  | 0.004 | |  | |  | 0.113 |
| California | Reference |  |  | | Reference | |  |  |
| Connecticut | 1.200 | 0.770-1.872 | 0.421 | | 1.116 | | 0.713-1.745 | 0.630 |
| Georgia | 1.202 | 0.870-1.661 | 0.264 | | 1.113 | | 0.803-1.540 | 0.519 |
| Hawaii | 1.348 | 0.708-2.566 | 0.364 | | 1.117 | | 0.583-2.138 | 0.738 |
| Iowa | 1.269 | 0.852-1.889 | 0.241 | | 1.224 | | 0.818-1.831 | 0.324 |
| Kentucky | 1.584 | 1.133-2.214 | 0.007 | | 1.526 | | 1.085-2.145 | 0.015 |
| Louisiana | 0.972 | 0.663-1.426 | 0.885 | | 1.006 | | 0.683-1.480 | 0.978 |
| Michigan | 0.692 | 0.417-1.149 | 0.155 | | 0.665 | | 0.399-1.106 | 0.116 |
| New Jersey | 0.873 | 0.603-1.265 | 0.474 | | 0.814 | | 0.560-1.180 | 0.277 |
| New Mexico | 2.536 | 1.528-4.209 | <0.001 | | 2.396 | | 1.440-3.987 | <0.001 |
| Utah | 0.852 | 0.433-1.675 | 0.642 | | 0.998 | | 0.506-1.965 | 0.995 |
| Washington | 0.967 | 0.597-1.566 | 0.891 | | 0.918 | | 0.565-1.489 | 0.728 |
